# Effect of experimental bleaching gels with polymers Natrosol and Aristoflex on the enamel surface properties

**DOI:** 10.1590/0103-6440202305248

**Published:** 2023-05-15

**Authors:** Iana Maria Costa Gonçalves, Danielle Ferreira Sobral-Souza, Antonio Carlos Roveda, Flávio Henrique Baggio Aguiar, Débora Alves Nunes Leite Lima

**Affiliations:** 1 Department of Restorative Dentistry, Piracicaba Dental School, University of Campinas - UNICAMP, P.O. BOX 52, 13414-903, Piracicaba, SP, Brazil.; 2 São Carlos Institute of Chemistry (ISQC), University of São Paulo (USP), 13566-590, São Carlos, SP, Brazil.

**Keywords:** Dental bleaching, raman spectroscopy, carbamide peroxide, thickener, dental enamel

## Abstract

Natrosol and Aristoflex^®^ AVC polymers are widely applied in the cosmetic industry and have recently been applied as a thickener option in the composition of dental bleaching gels, with the purpose to reduce the adverse effects on enamel mineral components. The aim of this study was to evaluate the color variation (ΔE^*^_ab_, ΔE_00,_ ∆WI_D_), surface roughness (Ra), and mineral content quantification (Raman Spectroscopy) of dental enamel after bleaching treatment with experimental gel-based on 10% carbamide peroxide (CP), containing Carbopol, Natrosol, and Aristoflex^®^ AVC. Sixty bovine teeth were randomly divided into 6 groups (n=10): Negative Control (**NC**) - no treatment; Positive Control (**PC**) - Whiteness Perfect 10% - FGM; CP with Carbopol (**CPc**); CP with Natrosol (**CPn**); CP with Aristoflex^®^ AVC (**CPa**); **NCP** - no thickener. Data were analyzed, and generalized linear models (∆WI_D_ -T_0_ x T_1_) were used for repeated measurements in time for Ra and with a study factor for ΔE^*^_ab_ and ΔE_00_. For the evaluation of the mineral content, data were submitted to *one-way* ANOVA and Tukey tests. For enamel topographic surface analysis the Scanning Electron Microscope (SEM) was performed. A significance level of 5% was considered. ΔE^*^_ab_ and ΔE_00_ were significantly higher for CPc, CPn, CPa, and NCP groups. (∆WI_D_) showed a significantly lower mean than the other groups for NC in T_1_. After bleaching (4-hour daily application for 14 days), Ra was higher in the CPc, CPn, and PC groups. For CPa, Ra was not altered. No significant difference was found in the quantification of mineral content. CPa preserved the surface smoothness more effectively. Aristoflex^®^ AVC is a viable option for application as a thickener in dental bleaching gels, presenting satisfactory performance, and maintaining the whitening efficacy of the gel, with the advantage of preserving the surface roughness of tooth enamel without significant loss of mineral content.

## Resumo

Os polímeros Natrosol e Aristoflex® AVC são amplamente utilizados na indústria cosmética e foram recentemente aplicados como uma opção de espessante na composição de géis de clareamento dental, com o objetivo de reduzir os efeitos adversos sobre os componentes minerais do esmalte. O objetivo deste estudo foi avaliar a variação de cor (ΔE*ab, ΔE_00_, ∆WID), rugosidade da superfície (Ra), e quantificação do conteúdo mineral (Raman Spectroscopy) do esmalte dentário após clareamento dental com gel experimental baseado em 10% de peróxido de carbamida (CP), contendo Carbopol, Natrosol e Aristoflex® AVC. Sessenta dentes bovinos foram divididos aleatoriamente em 6 grupos (n=10): Controle Negativo (CN) - sem tratamento; Controle Positivo (CP) - Brancura Perfeita 10% - FGM; CP com Carbopol (CPc); CP com Natrosol (CPn); CP com Aristoflex® AVC (CPa); NCP - sem espessante. Os dados foram analisados e modelos lineares generalizados (∆WID -T0 x T1) foram usados para medições repetidas no tempo para Ra e com um fator de estudo para ΔE*ab e ΔE00. Para a avaliação do conteúdo mineral, os dados foram submetidos a testes unidirecionais de ANOVA e Tukey. Para a análise da superfície topográfica do esmalte, o Microscópio Eletrônico de Varredura (SEM) foi realizado. Um nível de significância de 5% foi considerado. ΔE*ab e ΔE_00_ foram significativamente maiores para os grupos CPc, CPn, CPa e NCP. (∆WID) mostrou uma média significativamente menor do que os outros grupos para NC em T1. Após o clareamento (aplicação diária de 4 horas por 14 dias), Ra foi maior nos grupos CPc, CPn e PC. Para CPa, Ra não foi alterado. Nenhuma diferença significativa foi encontrada na quantificação do conteúdo mineral. O CPa preservou a suavidade da superfície de forma mais eficaz. Aristoflex® AVC é uma opção viável para aplicação como espessante em géis de clareamento dental, apresentando desempenho satisfatório e mantendo a eficácia clareadora do gel, com a vantagem de preservar a rugosidade da superfície do esmalte dentário sem perda significativa de conteúdo mineral.

## Introduction

Carbamide peroxide (CP - chemical formula CH_6_N_2_O_3_) is a bleaching agent widely used in supervised dental bleaching. During its interaction with saliva, this agent dissociates into hydrogen peroxide (HP - chemical formula H_2_O_2_) and urea (CH₄N₂O) ^1^ ^,^ ^2^. It is typically used at concentrations of 10-22% in this technique, equivalent to 3.5-7.3% HP ^3^. At-home bleaching treatment with 10% CP is considered the gold standard, primarily due to its efficiency and biosafety and because it is the only approach approved by the American Dental Association (ADA) ^(^ ^4^ ^,^ ^5^. Another bleaching agent used in this technique is HP, at concentrations of 3-10% ^(^ ^5^.

The mechanism of action of dental bleaching is based on the release of free radicals when in contact with the dental surface, which are diffused through the enamel. A redox reaction occurs, leading to the breakdown of long-chain chromogenic molecules into smaller ones ^3^. However, this procedure generates several side effects for the patient, such as dentin hypersensitivity, gingival irritations, and chemical and morphological changes on the dental enamel surface ^6^. It has been evidenced in clinical trials that the overall safety of peroxides can lead to potential complications such as teeth deproteinization and demineralization, including inorganic component loss, such as phosphate and carbonate ions ^(^ ^7^.

In addition to CP or HP, bleaching gels have thickeners in their composition, which are chemical components that provide these gels with viscosity ^(^ ^3^. These thickeners improve the maintenance of the gel under the dental surface, increasing contact with peroxide and, thus, prolonging the exposure time to these active compounds ^(^ ^8^. Carbopol and glycerin are the most commonly used thickeners. Nevertheless, deleterious effects such as alterations on surface roughness and reduction in microhardness have been reported ^9^ ^,^ ^10^ ^,^ ^11^ ^,^ ^12^ when using this polymer as a thickener. The demineralizing capacity of Carbopol can be attributed to its ability to chelate calcium ions, thus hindering the remineralization process provided by saliva ^10^.

Two new polymers have recently been proposed as bleaching thickeners, with the aim to reduce adverse effects on enamel mineral components: Natrosol and Aristoflex^®^ AVC ^26^. Natrosol is non-ionic and has the advantage of having a wide pH stability (pH 2.0-12.0) ^(^ ^8^, which can be used together with acidic compounds such as bleaching gels. This approach allows maximum control in the transformation of CP into free radicals. Furthermore, it has been shown that substituting Carbopol with Natrosol, led to smaller changes in enamel surface roughness ^(^ ^8^, justifying the use of this polymer as a thickener in CP bleaching agents ^(^ ^8^ ^,^ ^13^.

 Aristoflex^®^ AVC is a copolymer of sulphonic acid acryloyldimethyltaurate and vinylpyrrolidone, which, in contrast to Carbopol, is a pre-neutralized cationic synthetic polymer that presents high stability in acid pH, allowing the formation of gels with a good consistency ^(^ ^13^.

*In vitro* studies have evaluated the potential morphological changes in the dental surface after bleaching by scanning electron microscopy (SEM) ^(^ ^11^ ^,^ ^12^. However, to our best knowledge, structural changes at a molecular level have not been previously reported when using bleaching formulations associated with the polymers Natrosol and Aristoflex^®^ AVC; such analyses would allow a better understanding of bleaching gels clinical performance ^(^ ^14^.

In this context, Raman spectroscopy is an effective method to evaluate the effects of CP and/or HP on the dental structure ^(^ ^15^ ^,^ ^16^ ^,^ ^17^ ^,^ ^18^. This analysis involves investigating the mineral content via excitation of vibrational modes, therefore, being considered an ideal method to examine dental structure inorganic contents, such as phosphate and carbonate molecules ^(^ ^14^ ^,^ ^15^ ^,^ ^16^.

It is necessary to study bleaching gel component modifications to a molecular level with the purpose to reduce the adverse effects on enamel mineral components. Therefore, this study aimed to evaluate the alteration of enamel inorganic components relative to phosphate and carbonate ions, after bleaching treatment with 10% CP, containing Natrosol and Aristoflex^®^ AVC as thickeners, as well as the physical properties of color and surface roughness. The null hypotheses tested were: 1) that the experimental bleaching gels would not affect the color variation after bleaching treatment, 2) the physical property of surface roughness would not be altered after bleaching treatment, and 3) the use of polymers Natrosol and Aristoflex^®^ AVC as thickeners, would not affect the mineral content of dental enamel after bleaching treatment.

## Material and methods

### Sample Preparation

A total of 60 bovine teeth were sectioned to obtain enamel/dentin specimens with dimensions of 4 x 4 x 3 mm (1 mm enamel and 2 mm dentin) using a double-sided diamond disc section (Extec 4" x 0.12 x 1/2) coupled to a precision metallographic cutter (Isomet 1000, Buehler).

Enamel surfaces were flattened with silicon carbide (SiC) sandpapers (600-grit, 1200-grit, 2500-grit, and 4000-grit) in crescent order, under constant water irrigation, using a rotative grinder polisher (Arotec Ind. Com., Cotia, SP, Brazil). To complete the polishing procedure, 1 μm, and 1/4 μm diamond paste and felt discs (Arotec Ind. Com.) The specimens were stored in closed plastic containers, covered with absorbent paper wetted in deionized water, and stored in a refrigerator at 4°C and received a demarcation on one of the lateral surfaces with a 1014 spherical diamond tip (KG Sorensen, Barueri, SP, Brazil) to standardize the initial and final analysis for color and roughness. The specimens were randomly divided into six groups (n=10) according to the bleaching treatment (Box 1). G*Power program was used for sample calculation, with a large effect size (f=0.38) and power of at least 80% (β=0.20) for the main effects: group and time, and the interaction (group x time), with a significance level of 5% (α=0.05).

### Staining Protocol

To ensure a similar staining effect, the surfaces of the specimens were protected with acid-resistant varnish, with exception of the enamel surface (Risqué Colorless, Taboão da Serra, Brazil). A 1.8 g black tea solution was prepared (Dr. Oetker LTDA, São Paulo, SP, Brazil) in 100 ml of distilled water boiled for 3 minutes, and infused for 5 minutes. The dental fragments were immersed in this solution, which was replaced every 24 hours for 6 days. After this immersion period, the samples were stored in artificial saliva (composition: Ca 1.5 mmol/L; P 0.9 mmol/L; KCl mmol/L; 0.1mol/L tris buffer solution) adjusted to pH 7.0 at 37ºC (±1ºC) ^(^ ^12^ ^,^ ^19^ for 1 week; during this period, saliva exchange was performed daily to stabilize the color ^(^ ^20^. Previously to color reading, the black tea lees formed on the enamel and dentin were removed through prophylaxis, using a rubber cup and a mixture of ultrafine granulation pumice stone and water (2:1), at a low rotation for 30 seconds on each specimen face. To return the polishing and brightness of the specimens surface after this procedure, a 4000-grit silicon carbide sandpaper (SiC) were used.

**Box 1:**
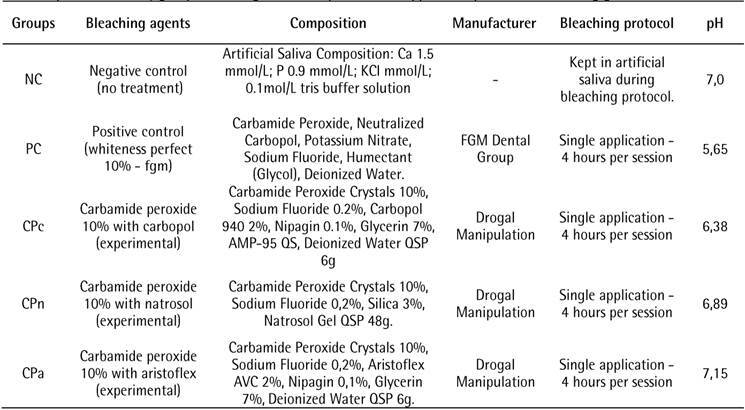
Experimental study groups according to the composition and application protocol of bleaching gels.

### Bleaching Protocol

Prior to bleaching treatment, self-curing acrylic resin devices (JET, São Paulo, SP, Brazil) were made for specimen allocation. In the center of the device, 5 x 5 mm silicone blocks (Elite HD + normal setting-© Zermack SpA- Badia Polesine (RO), Italy) were positioned. After resin polymerization, the silicone blocks were removed, and the specimens were fixed in the center of the devices with sticky wax (ASFER indst. Química Ltda, São Caetano do Sul, São Paulo, SP, Brazil), avoiding direct contact with dentin, once the gel penetrates the substrate through diffusion. The devices were fixed with the aid of orthodontic wire (Elastic Orthodontic Wire CrNi Redondo 0.70 mm., Sorocaba, São Paulo, SP, Brazil) in individual containers ^(^ ^10^.

Bleaching treatment was performed for 14 consecutive days with 4 hours of daily application. The negative control was not submitted to any kind of exposure and the solution of artificial saliva was replaced each day. During the performance of this protocol, the specimens were kept at a constant relative humidity and controlled temperature (37ºC±2ºC). After completing the daily bleaching protocol, the bleaching gel was removed with flexible cotton stems, and each specimen was carefully washed with distilled water to remove gel remnants. Then, the specimens were stored in 2.0 mL of artificial saliva, which was renewed daily throughout the experiment.

### Color Measurements (∆E^*^_ab_ and ∆E_00_)

Color analyses were performed before and 24 hours after the bleaching treatment. The specimens were placed in a Teflon device inside a light chamber (GTI Mini Matcher MM1e, GTI Graphic Technology Inc., Newburgh, NY, USA) to standardize the ambient light. A reflectance spectrophotometer (Konica Minolta CM-700d) was previously calibrated according to the manufacturer's instructions ^(^ ^12^. The overall color variation was analyzed using the CIELAB (∆E^*^_ab_), CIEDE 2000 (∆E_00_) and Whiteness Index (ΔWI_D_) systems. For the CIEDE 2000 (∆E_00_), the specimens were read in triplicate and based on the following equation:

$${∆E}_{00}={\left[{\left(\frac{∆L'}{KLSL}\right)}^{2}+{\left(\frac{∆C'}{KCSC}\right)}^{2}+{\left(\frac{∆H'}{KHSH}\right)}^{2}+RT\left(\frac{∆C'}{KCSC}\right)\;\left(\frac{∆H'}{KHSH}\right)\right]}^{1/2}$$

Where, ∆L', ∆C', and ∆H' refer to luminosity (value), chroma and hue, and RT (rotation function) is responsible for the interaction between chroma and hue differences in the blue region. The weighting functions (SL, SC, and SH) refer to the adjustment of the total color difference, and parametric factors (KL, KC, and KH) are terms of correction for experimental conditions ^(^ ^21^ ^,^ ^22^.

For the CIELAB system, each sample was read in triplicate, and the mean obtained was quantified in three coordinates (L*, a*, b*), which define the color of an object within a 3D color space through the software OnColor QC Lite (Konica Minolta, Japan). The L* coordinate represents the degree of luminosity ranging from 0 (black) to 100 (white), coordinate a* determines the variation in the red (a*+) and green (a*-) axis, and coordinate b* represents the variation in the yellow (b*+) and blue (b*-) axis. For the CIELAB system, the overall color change was calculated using the equation: ΔE^*^_ab_ = [(L_1_ - L_0_)^2^ + (a_1_ - a_0_)^2^ + (b_1_ - b_0_)^2^]^1/2^ (Commission Internationale de l'Eclairage 1978) ^22^.

The Whitening Index (ΔWI_D_) was calculated based on the following equation:

ΔWI_D_=0.511ΔL* - 2.324Δa* - 1.100Δb*

The L* coordinate was considered for sample allocation after the initial reading, in which the samples that presented very high (dark) or very low (light) values were excluded from the study ^(^ ^19^.

### Surface Roughness (Ra)

The surface roughness of the specimens was measured previously to bleaching protocol and subsequently, 24 hours after exposure to bleaching gels. A surface roughness measuring machine (Mitutoyo SJ-410, São Paulo, SP, Brazil) was used. Three scans were performed, in a 3.0 mm extension, with the reading tip always passing through the geometric center of the specimen, and the change of position allowed by the 120º rotation at the base. The counterclockwise direction was standardized for all readings, and the first reading was established in the surface previously demarcated for color reading standardization. Thus, Ra was calculated through the average between the peaks and valleys recorded, and the means of the three scans were considered.

### Raman Spectroscopy

**Figure 1:**
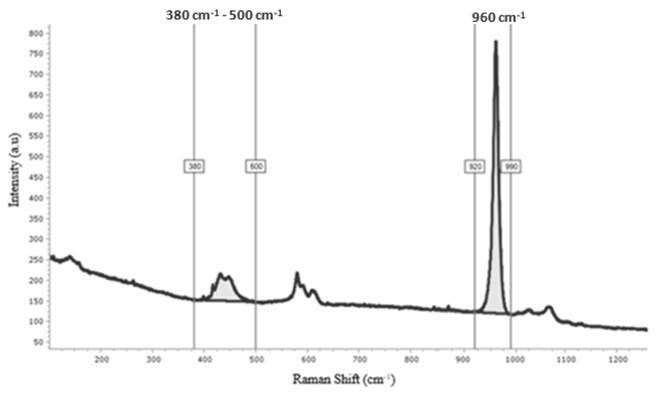
Comparison ratio between the area of interest and phosphate 960 cm^-1^ (PO_4_^-3^) peak.

After bleaching treatment, the inorganic concentration of phosphate and carbonate ions were analyzed using a Raman Spectroscopy (Raman LabRAM HR Evolution spectrometer - Horiba), with the following specifications: laser with a wavelength of 785 nm, Edge 1800 (500nm) diffraction grid, objective lens with x100 increase, 64 scans, acquisition time of 2 seconds, and analysis range from 200 to 2000 cm^-1 (^ ^15^ ^,^ ^18^. Prior to Raman analysis, specimens were submitted to an ultrasonic machine (Marconi, Piracicaba, SP, Brazil) for 15 minutes to remove debris, and subsequently stored in deionized water. The frequency of the spectrum was established considering the variation of 200-2000 cm^-1^. For analysis of enamel inorganic components, the interest peaks analyzed were: 430-449 cm^-1^ (PO_4_^-3^ - *v2)*, 580-611cm^-1^ (PO_4_^-3^ - *v4*, and 960 cm^-1^ (PO_4_^-3^ - *v1)*, referring to the vibrational modes of phosphate ^17^ ^,^ ^18^ and 1043-1070 cm^-1^ (CO_2_^-3^ - *v3*) related to the carbonate vibrational mode ^17^ ^,^ ^18^ ^,^ ^24^. To determine the area of the vibrational bands, the spectra were corrected in relation to the baseline and normalized at the peak of 960 cm^-1^ (PO_4_^-3^) ^16^ ^,^ ^17^, then a ratio between the area for the peak of 960 cm^-1^ (PO_4_^-3^) and the areas referring to phosphate and carbonate *v2, v4* and *v3* was measured (Figure 1). To obtain the area of each vibrational mode, Gaussian shapes performed the band decomposition, and the spectra obtained were evaluated using analytical software (SpectraGryph^®^ Optical Spectroscopy Software, ^©^2016: Dr. Friedrich Menges Software-Entwicklung, Oberstdorf, Germany).

### Statistical Analysis

 The data were analyzed with the R program (R Core Team (2021). R: A language and environment for statistical computing. R Foundation for Statistical Computing, Vienna, Austria). Next, generalized linear models with repeated measurements in time for roughness and with a study factor for ∆E^*^_ab_ and ∆E_00_ were used. For ∆WI_D_, generalized linear models were used to analyze the effect of groups in T_0_ (before bleaching) and T_1_ (24 hours after bleaching). For surface roughness (Ra), the analysis was carried out according to the following factors: bleaching treatment (six levels: NC, PC, CPc, CPn, CPa, NCP) and time (baseline and after 24 hours of the bleaching treatment).

The values of Carbonate and Phosphate ratios were submitted to the normal distribution test (Shapiro Wilk), and the Negative Control (NC) was analyzed by paired T-test and compared to the other groups by *one-way* ANOVA followed by Tuckey test with 95% confidence, according to 6 bleaching treatment groups (NC, PC, CPc, CPn, CPa, NCP), considering the factor presence/absence of thickener. A significance level of 5% was set for all analyses, and Minitab^®^ Statistical Software (^©^2021. Minitab, LLC. USA) was used.

### Scanning Electron Microscope (SEM)

For topographical evaluation of dental enamel surface and morphology, 4 specimens were randomly selected. Specimens were previously metalized (Bal-Tex SCD 050 sputter coater, Germany) with a thin layer of gold alloy. Photomicrographs of representative areas were obtained in a Scanning Electron Microscope (Jeol, JSM 5600LV, Tokyo, Japan) under a 4000x magnification, with 15 kV.

## Results

Table 1 shows that the variation in color (∆E^*^_ab_ and ∆E_00_) was significantly higher in the experimental groups CPc, CPn, Cpa, and NCP than in the NC and PC groups (p<0.0001). The PC group showed increased color variation than the NC group (p<0.0001). There was no significant difference between the experimental groups with different thickeners (CPc, CPn, CPa) and also without thickener (NCP) (p>0.05).

**Table 1:** Color variation (∆E^*^_ab_, ∆E_00_) (standard deviation), median (minimum and maximum value) according to the group.

Group	Color Variation			
	∆E^*^_ab_		∆E_00_	
	Mean (standard deviation)	Median (minimum and maximum value)	Mean (standard deviation)	Median (minimum and maximum value)
No treatment (NC)	2,10 (0,82) c	1,99 (0,70; 3,37)	1,68 (0,65) c	1,57 (0,54; 2,72)
Whiteness Perfect 10% - FGM (PC)	8,74 (3,18) b	8,99 (4,06; 14,46)	6,57 (2,25) b	6,77 (3,08; 10,66)
Carbamide Peroxide 10% + Carbopol thickener (CPc)	11,56 (2,83) a	12,04 (6,21; 14,79)	8,78 (2,14) a	8,91 (4,95; 11,15)
Carbamide Peroxide 10% + Natrosol thickener (CPn)	12,27 (2,23) a	11,90 (9,49; 16,30)	9,02 (1,52) a	8,99 (6,99; 11,67)
Carbamide Peroxide 10% + Aristoflex thickener (CPa)	11,76 (4,12) a	11,12 (6,40; 19,44)	8,88 (2,88) a	8,49 (4,91; 14,05)
Carbamide Peroxide 10% (no thickener) (NCP)	12,64 (3,6) a	12,96 (8,45; 20,90)	9,29 (2,36) a	9,64 (6,17; 14,66)
p-value	<0,0001		<0,0001	

*Distinct letters (lowercase letters horizontally) indicate significant differences (p≤0.05).

Table 2 shows that there was no significant difference between the groups regarding ∆WI_D_/T_0_ (p=0,9134). Regarding ∆WI_D_/T_1_, the NC group presented a significantly lower mean than the other groups submitted to bleaching treatment (p<0.0001).

**Table 2:** Mean (standard deviation), median (minimum and maximum value) of ∆WI_D_ variation according to the group.

Group	∆WI_D_/T_0_		∆WI_D_/T_1_	
	Mean (standard deviation)	Median (minimum and maximum value)	Mean (standard deviation)	Median (minimum and maximum value)
No treatment (NC)	15,57 (4,69) a	16,24 (5,56; 22,04)	13,33 (4,72) b	12,97 (4,69; 20,58)
Whiteness Perfect 10% - FGM (PC)	17,14 (4,62) a	16,29 (11,87; 25,29)	29,78 (2,30) a	29,45 (27,14; 34,73)
Carbamide Peroxide 10% + Carbopol (CPc)	16,14 (3,83) a	15,06 (12,03; 24,03)	32,87 (1,81) a	32,55 (30,66; 35,31)
Carbamide Peroxide 10% + Natrosol (CPn)	15,88 (4,01) a	17,37 (8,30; 20,71)	33,21 (2,36) a	33,13 (28,88; 37,08)
Carbamide Peroxide 10% + Aristoflex (CPa)	16,86 (4,75) a	18,09 (7,96; 22,27)	33,65 (3,64) a	33,77 (29,26; 38,33)
Carbamide Peroxide 10% (no thickener) (NCP)	15,08 (4,55) a	15,61 (7,60; 24,15)	32,68 (4,61) a	33,99 (23,6; 38,61)
p-value	0,9134		<0,0001	

*Distinct letters (lowercase letters horizontally) indicate significant differences (p≤0.05).

Table 3 demonstrates significant interactions between groups and times for roughness (p<0.0001). Ra increased significantly in the PC (positive control), CPc, and CPn groups, 24 hours after bleaching. NC (negative control), CPa, and NCP (no thickener addition) showed lower roughness values than the other groups, and no significant difference was found between them (p<0.0001) (Table 2). CPc showed the highest values of surface roughness (p>0.05) (Table 2).

**Table 3:** Mean roughness (standard deviation), median (minimum and maximum value) according to group and time.

Group	Before bleaching		24 hours after bleaching	
	Mean (standard deviation)	Median (minimum and maximum value)	Mean (standard deviation)	Median (minimum and maximum value)
No treatment (NC)	0,03 (0,01) Aa	0,03 (0,02; 0,05)	0,03 (0,01) Ad	0,03 (0,02; 0,05)
Whiteness Perfect 10% - FGM (PC)	0,03 (0,01) Ba	0,03 (0,02; 0,05)	0,12 (0,01) Ac	0,11 (0,10; 0,14)
Carbamide Peroxide 10% + Carbopol (CPc)	0,03 (0,01) Ba	0,03 (0,01; 0,06)	0,17 (0,03) Aa	0,18 (0,12; 0,20)
Carbamide Peroxide 10% + Natrosol (CPn)	0,03 (0,01) Ba	0,03 (0,02; 0,05)	0,14 (0,01) Ab	0,14 (0,13; 0,16)
Carbamide Peroxide 10% + Aristoflex (CPa)	0,03 (0,01) Aa	0,03 (0,02; 0,05)	0,03 (0,01) Ad	0,03 (0,02; 0,05)
Carbamide Peroxide 10% (no thickener) (NCP)	0,03 (0,01) Aa	0,03 (0,02; 0,05)	0,04 (0,01) Ad	0,04 (0,02; 0,05)

*Distinct letters (capital letters horizontally and lowercase letters vertically) indicate significant differences (p≤0.05). p(group)=0.0021; p(time)<0.0001; p(interaction)<0.0001.

**Figure 2:**
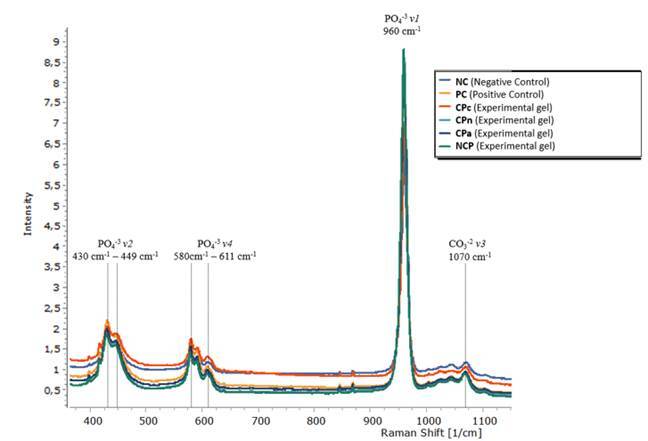
Raman average spectra of mineral components in the 400-2,000 cm^-1^ region showing the comparison between phosphate (*v1* 960 cm^-1^, *v*2, 430 - 449 cm^-1^ and *v*4, 580 - 611 cm^-1^) and carbonate *v*3 (1070 cm^-1^) vibrational modes peaks. *NC (Negative Control/No treatment); PC (Positive Control - Whiteness Perfect 10% FGM); CPc (Experimental gel based on CP 10% + Carbopol); CPn (Experimental gel based on CP 10% + Natrosol); CPa Experimental gel based on CP 10% + Aristoflex); NCP (Experimental gel based on CP 10% without thickener addition). ** p-value = 0,307 v2 (PO_*4*_^*-3*^ *); p = 0,349 v4 (PO*_*4*_^*-3*^ *); p = 0,921 v3 (CO*_*3*_^*-2*^ *).*

Figure 2 shows the results for the spectra of mineral components obtained in the 400-2000 cm^-1^ region comparing the peaks of vibrational modes for phosphate (*v2*, 430-449 cm^-1^ and *v4*, 580-611 cm^-1^) and carbonate *v3* (1043-1070 cm^-1^). No significant variations were found for the groups treated with different thickeners, p=0,307 (*v2*, 430-449 cm^-1^; p=0,349 (*v4*, 580-611 cm^-1^); p=0,921 (*v3* 1070 cm^-1^).

**Figure 3:**
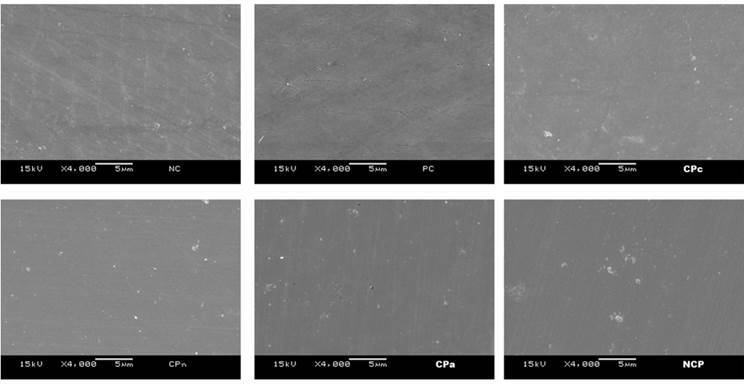
Photomicrographs obtained by SEM; ***NC (Negative Control/No treatment); PC (Positive Control - Whiteness Perfect 10% FGM); CPc (Experimental gel based on CP 10% + Carbopol); CPn (Experimental gel based on CP 10% + Natrosol); CPa Experimental gel based on CP 10% + Aristoflex); NCP (Experimental gel based on CP 10% without thickener addition). All groups maintained the surface polishing, but CPa showed more smoothness in relation to the groups treated with other experimental bleaching gels.***

The Images of SEM allowed to observe that the surface of enamel treated with experimental bleaching gels maintained the polishing of the surface, provided by the use of SiC sandpaper. In all treated groups and in the CN group, the mineral content was not significantly altered. Thus, none of the groups presented demineralization (Figure 3).

## Discussion

Dental enamel color was assessed according to the CIE system (L* a* b*) and CIEDE 2000, which quantifies the general color variation (ΔE^*^_ab_ - ΔE_00_) ^21^ ^,^ ^22^. ΔE^*^_ab_ parameter provides information about the overall color change of an object, considering thresholds of perceptibility (1.2) and acceptability (2.7) ^(^ ^21^ ^,^ ^22^. The scientific literature states that a perception threshold in color variation occurs when ΔE^*^_ab_ equals 3 ^10^ ^,^ ^21^. The use of ∆E_00_ parameter has become quite popular in recent years in dentistry due to the corrections in the formula referring to chroma and hue patterns and the rotation function (RT) responsible for the interaction between chroma and hue differences in the blue region. For ΔE_00_, the thresholds of perceptibility and acceptability are also considered and are established in the literature as 0.8 and 1.8, respectively ^21^ ^,^ ^22^.

The first null hypothesis was rejected since the bleaching gels used in this study affected the color variation after bleaching treatment. However, there was no significant difference between the values for CIELAB (∆E^*^_ab_) and CIEDE 2000 (∆E_00_) systems. The results for the Whiteness Index (∆WI_D_) - T_1_, showed a significantly lower mean than the other groups for NC, which was expected once all the other groups were submitted to bleaching treatment (Table 2).

In this study, the specimens were previously stained with a black tea solution to standardize their initial color ^19^. Reduction in the reddish-brown coloration demonstrated the bleaching method’s effectiveness ^25^. The variation in color (∆E^*^_ab_ and ∆E_00_) (Table 1) was significantly higher in the experimental groups containing the thickeners Carbopol, Natrosol, and Aristoflex than in the negative and positive controls. The values found for general color variation related to ΔE^*^_ab_ and ΔE_00_ were above the values of perceptibility and acceptability, confirming the bleaching efficacy of the gels analyzed. These results agree with those in the literature ^(^ ^8^, showing that the color variation of CP presented higher ΔE^*^_ab_ than nonbleached groups, irrespective of the thickener used.

Although all bleaching treatments used were effective in achieving positive results for bleaching, the experimental gels, including the one without the addition of thickener in its composition, showed higher color variation for both ΔE^*^_ab_ and ΔE_00_ than the positive control (Whiteness Perfect 10% - FGM) in the same period of time (Table 1). This finding can be explained based on the thickener´s properties. Carbopol is the most commonly used thickener in commercial bleaching gels, though it is ionic and has low pH stability ^8^ ^,^ ^13^, its efficacy when associated with 10% CP has been demonstrated ^9^. The different thickeners tested in the present study did not affect the CP bleaching performance. The goal of adding a thickener to a CP bleaching gel is to provide closer contact with the tooth surface and prolong free radical release. Moreover, the thickener is responsible for turning the liquid bleaching agents into a gel, providing the gel viscosity, essential to guarantee effective contact between the dental surface and the bleaching gel ^8^ ^,^ ^10^ ^,^ ^13^.

Aristoflex^®^ AVC effectively reduced the loss of minerals compared to the use of a gel containing Carbopol as a thickener (positive control), causing a lower impairment to the physical properties of dental enamel ^(^ ^13^. It has been well-accepted in the literature that HP has unspecific action within the dental structure ^(^ ^3^ ^,^ ^8^. Consequently, enamel demineralization during a bleaching treatment can be attributed to the mechanism of action of HP, provided through the reaction involving the releasing of CP and the association with different thickeners. It is important to note that the experimental gels analyzed, as well as in the positive control, contain sodium fluoride (NaF) in its formulation, and the addition of this component can influence mineral loss. The second null hypothesis was rejected once Natrosol affected the surface roughness (Ra), while Aristoflex^®^ AVC did not cause significant changes.

The results for Ra were similar to previous results ^(^ ^13^, in which groups treated with CPc had higher roughness values compared to groups using Natrosol and control groups. The results presented in this study are also in agreement with Gouveia et al. ^(^ ^13^, who showed no significant differences after the treatments with CPa and NCP (no thickener), and the control group maintained their initial profile values. Also, the group treated with the PC (positive control) presented the highest roughness values compared the to control group.

For Natrosol, there was an increase in surface roughness after the bleaching treatment. Nevertheless, this increase was lower compared to the experimental group containing Carbopol as a thickener and similar to that found when using the positive control gel. This increase in roughness did not occur with the use of Aristoflex^®^ AVC, in which the roughness remained unaltered, similar to the negative control.

As previously discussed, Carbopol (Carboxypolymethylene) contains water-soluble polymers used as a gelling agent in aqueous systems and emulsions, thus promoting solution viscosity ^8^ ^,^ ^10^ ^,^ ^13^. In agreement with the findings presented in this paper, the literature suggests that the changes in the dental surface can be attributed mainly to Carbopol’s low pH and high viscosity. Studies have shown that this thickener, derived from a carboxylic acid, has an acidic pH and may contribute to dental demineralization and alterations in surface roughness ^8^ ^,^ ^10^. For the incorporation of a viscosity agent into a bleaching product formulation, it must be buffered at a neutral pH acting as a compound avoiding negatively influence on the bleaching reaction ^(^ ^8^ ^,^ ^10^.

The enamel surface roughness was not altered after applying the bleaching treatment with Aristoflex^®^ AVC (Table 3). This finding can be explained because of a weakly formed film with the enamel surface. Due to its cationic nature, this polymer may attract fluoride ions, which are available in enamel, to bind to its positive sites, further decreasing the number of possible connections between the polymer and the dental structure, consequently reducing the formation of this film but allowing the dental surface to be free for salivary remineralization ^(^ ^10^ ^,^ ^13^.

Raman spectroscopy can be an effective method for evaluating the effects of CP and HP on the dental structure ^2^ ^,^ ^15^ ^,^ ^16^ ^,^ ^17^. Data on mineral content are obtained by investigating their energy via the excitation of vibrational modes. The so-called Raman scattering, also referred to as inelastic scattering, occurs when this vibrational energy is observed as additional peaks in the scattered light spectrum. These peaks are typical of the molecules of the compound that is under analysis and produces a kind of "fingerprint". The intensity of these points generates data regarding the concentration of specific groups present in the hydroxyapatite molecule ^14^ ^,^ ^15^ ^,^ ^16^ ^,^ ^17^ ^,^ ^18^.

In this research, no significant differences were found between the groups tested, and the peaks for phosphate and carbonate were not significantly changed after the bleaching treatment (Figure 2). Therefore, CP associated with different thickeners did not demineralize enamel to a significant degree. Thus, the third null hypothesis was accepted because none of the thickeners analyzed in this study significantly altered the mineral content of dental enamel (Figure 2). The use of Natrosol and Aristoflex^®^ AVC as thickeners can be as effective as Carbopol in ensuring that the inorganic content of enamel unalteres, with the advantage that Aristoflex^®^ AVC can reduce negative effects on dental enamel surface roughness properties.

 Phosphate and carbonate content may indicate the degree of enamel mineralization. The intensity of PO_4_^-3^ and CO_3_^-2^ in Raman spectroscopy is linearly proportional to the concentrations of phosphate and carbonate within hydroxyapatite molecules. Cavalli et al. ^(^ ^18^ found no differences before and after at-home bleaching with some commercial gels containing 10% CP. Controversially, Bistey et al. ^(^ ^7^ and Berger et al. ^16^ found morphological alterations in human enamel after different in-office bleaching protocols; the severity of enamel alteration was found to be related to the treatment time, peroxide concentration, and treatment protocol.

Samples were also stored in artificial saliva, a remineralizing solution used to simulate a clinical situation. This solution contains a high level of phosphate and calcium, which can create an ideal environment for mineral recovery ^17^. The modulator effect of saliva on the demineralization and remineralization process of the mineral content has been previously documented ^17^ ^,^ ^18^. Consequently, the remineralizing effect of artificial saliva was effective in promoting the deposition of minerals on the enamel surface. Furthermore, it is important to correlate the remineralizing potential of artificial saliva with the low concentration of the bleaching agent used (10% CP) and the incorporation of remineralizing agents in bleaching gels, such as sodium fluoride (NaF). The potential for remineralization must be acknowledged once the artificial saliva might have had a synergistic effect, increasing phosphate and carbonate concentrations, as well as the addition of sodium fluoride (NaF), which can influence the inorganic content incorporation, such as phosphate and carbonate ions.

Therefore, both Natrosol and Aristoflex^®^ AVC can be indicated in substitution to Carbopol since they presented bleaching efficacy similar to the treatment with Carbopol. Aristoflex® AVC effectively maintained bleaching efficacy, preserving the superficial enamel roughness and the inorganic content, additionally providing the ideal viscosity needed by a bleaching gel. Under the conditions of this *in vitro* study, Raman Spectroscopy analysis showed that the association of CP with Carbopol, Natrosol, and Aristoflex^®^ AVC preserved the enamel structure in relation to the molecular composition.

*In vitro* studies have evaluated the potential morphological changes in the dental surface after bleaching by scanning electron microscopy (SEM) ^11^ ^,^ ^12^. All groups maintained polishing of the enamel surface. However, it is possible to observe that the PC, CPc and CPn groups maintained a similar pattern, justified by the increase of Ra in these groups. Similarly, CPc presented a more irregular surface compared to the other groups, while CPa demonstrated a smoother surface (Figure 3). Nevertheless, further *in vivo* research should be performed to confirm whether these results are consistent in more reliable clinical conditions. The experimental bleaching gels with Natrosol and Aristoflex^®^ AVC presented similar bleaching efficacy to the one containing Carbopol, and all the experimental groups demonstrated higher color variation than the PC group (Positive Control) in the same period of time. Also, the experimental bleaching gels accomplished the objective of conserving the mineral content of dental enamel after bleaching with 10% CP. Aristoflex^®^ AVC provided the satisfactory performance of a bleaching gel, with the advantage of maintaining the smoothness of the enamel surface, with the lowest values for surface roughness.
